# Galectin-3 activates TLR4/NF-κB signaling to promote lung adenocarcinoma cell proliferation through activating lncRNA-NEAT1 expression

**DOI:** 10.1186/s12885-018-4461-z

**Published:** 2018-05-22

**Authors:** Wu Zhou, Xing Chen, Qinghua Hu, Xuliang Chen, Yingji Chen, Lingjin Huang

**Affiliations:** Department of Cardiothoracic Surgery, Xiangya Hospital, Central-South University, Xiangya road 87, Changsha, 410078 Hunan China

**Keywords:** Galectin-3, TLR4, Lung adenocarcinoma, Proliferation, lncRNA-NEAT1

## Abstract

**Background:**

Lung cancer remains the top contributor to cancer-related mortality worldwide. Long non-coding RNAs (lncRNAs) have been reported to participate in normal development and tumorigenesis. LncRNA nuclear enriched abundant transcript 1 (NEAT1) is highly expressed in lung cancer and promotes lung cancer cell proliferation and migration. However, the upstream regulatory mechanism still needs investigation.

**Methods:**

In the present study, we investigated the upstream regulators and mechanisms of NEAT1 expression disorders. We first examined NEAT1 expression in lung adenocarcinoma tissues and its correlation with clinic features in patient with lung adenocarcinoma; next, the detailed function of NEAT1 in lung cancer cell proliferation and migration was assessed. To investigate whether NF-κB acts as a transcription factor of NEAT1 to activate its expression, we validated the combination between NF-κB and NEAT1, and NF-κB regulation of NEAT1 upon LPS stimulation. Further, the effect of NF-κB upstream regulator, TLR4, on NEAT1 expression upon LPS stimulation was examined. Galectin-3 reportedly serves as a ligand of TLR4 and promotes TLR4, MyD88 and p-p65 expression; we investigated whether Galectin-3 could modulate lung adenocarcinoma cell proliferation and migration through TLR4/NF-κB/NEAT1. Finally, the expression and correlation of the above factors in lung adenocarcinoma tissues was validated.

**Results:**

NEAT1 is highly expressed in lung adenocarcinoma tissues and promotes lung cancer cell proliferation and migration. NF-κB binds to NEAT1 promoter to activate NEAT1 expression after LPS-stimulated p65 nucleus translocation. LPS stimulation activates TLR4 signaling, followed by downstream NF-κB activation, and ultimately NEAT1 expression activation. Galectin-3 activates TLR4 signaling thus affecting lung cancer cell proliferation and migration through TLR4/NF-κB/NEAT1. Galectin-3 and TLR4 expression are abnormally up-regulated in lung adenocarcinoma tissues, and positively correlated with NEAT1 expression.

**Conclusion:**

We confirmed that Galectin-3 as a ligand of TLR4 induced TLR4 signaling activation in lung adenocarcinoma cells, thereby activating downstream p65 nucleus translocation, promoting NEAT1 expression, and finally affecting lung adenocarcinoma cell proliferation and migration. Inhibiting Galectin-3-induced TLR4 signaling activation, thus to reduce p65-activated NEAT1 expression might be a promising strategy of suppressing lung adenocarcinoma cell proliferation and migration.

**Electronic supplementary material:**

The online version of this article (10.1186/s12885-018-4461-z) contains supplementary material, which is available to authorized users.

## Background

Lung cancer has been regarded as a main cause of cancer incidence and cancer-leading mortality worldwide; less than 20 % patients with lung cancer obtain an overall survival > 5 years [1, 2]. Lung adenocarcinoma, rising as the main histological type of lung cancer due to changes in lifestyle such as smoking habit, accounts for approximately up to 50% of lung cancer cases [3]. Unfortunately, most lung adenocarcinoma patients treated with standard cytotoxic chemotherapy ultimately develop drug-resistance. Developing more effective strategies or targeted agents can be of great clinic value for lung adenocarcinoma treatment.

It is well-known that over 90 % of the human DNA could be transcribed with up to 2% of them encode protein; this part of transcripts that do not encode protein are called non-coding RNAs (ncRNAs) [4, 5]. Long non-coding RNAs (lncRNAs) participate in normal developmental and pathological processes, including cancers [6]. For example, lncRNA NEAT1 facilitates pancreatic cancer progression through negatively modulating miR-506-3p [7]. In breast cancer, NEAT1 interacts with miR-101 to modulate cancer cell proliferation through EZH2 [8]. In lung cancer, NEAT1 acts as an oncogenic lncRNA through affecting cancer cell proliferation, invasion and migration via different downstream signaling pathways [9–11]. More importantly, NEAT1 expression is dramatically upregulated in plasma samples of patients with NSCLC (non-small cell lung cancer); higher NEAT1 expression may be related to tumorigenesis and progression of NSCLC, suggesting the application of NEAT1 in personalized targeted therapy [12, 13]. Investigating the upstream regulators and mechanisms of NEAT1 expression disorders may provide a novel perspective to modulate lung adenocarcinoma cell hyperproliferation and migration.

NF-κB is a protein complex which can affect DNA transcription, cytokine release and cell survival [14]; it has been regarded as a key transcription factor which is constitutively activated in many cancers [15–18]; NF-κB activation is a crucial contributor in cancer progression [19, 20]. Commonly, NF-κB activity is shut down by IκB binding NF-κB complex to prevent the nucleus translocation, thus maintaining an inactive state of NF-κB [17–19]. Cell stimulation including pro-inflammatory factors TNF-α, IL-1 and bacterial lipopolysac-charide (LPS) disrupts this dynamic balance between cytosolic and nuclear localization, leading to NF-κB nucleus translocation and downstream gene expression which may contribute to cancer cell survival, growth and metastasis [21]. Interestingly, aberrant NF-κB activation commonly happens in many malignant tumors, including lung cancer [22, 23]. Whether NF-κB can be aberrantly activated by upstream regulators, thus activating NEAT1 expression in lung adenocarcinoma still remains unclear.

Here, we explored the upstream regulatory factors and mechanisms of NEAT1 abnormal overexpression in lung adenocarcinoma, and further suggest a regulatory path formed by Galectin-3, TLR4, NF-κB and NEAT1 that may contribute to the hyperproliferation and migration of lung adenocarcinoma cells.

## Methods

### Tissues and cell lines

Eighty-three cases of lung adenocarcinoma tissues and matched non-cancerous tissues were collected from patients who accepted surgery at the authors’ institution under the approval of Ethic Committee of the institution. These specimens were sent for routine pathological evaluation or instantly frozen in LN (liquid nitrogen).

Two human lung cancer cell lines, A549 (CCL-185™) and H1299 (CRL-5803™) were purchased from ATCC (Manassas, VA, USA) and cultured in RPMI 1640 with 10% FBS (Gibco®, Waltham, MA, USA). For LPS and CLI-095 treatment, 20 ng/ml LPS (Sigma, St. Louis, MI, USA) or 50 nmol/L CLI-095 (Invitrogen, Waltham, MA, USA) was used to stimulate the target cells for 4 h, then cells were harvested for further experiments.

### Cell transfection

The suppression of NEAT1 and p65 expression was achieved by transfection of si-NEAT1 or si-p65 (Genepharma, Shanghai, China) with Lipofectamine® 2000 agent (Invitrogen). A pcDNA3.1/p65 was used to overexpress p65 expression. Transfection of pcDNA3.1/Galectin-3 or pLVX/sh-Galectin-3 was performed to overexpressing or knocking down Galectin-3 (GeneCopoecia, Guangzhou, China).

### Real-time PCR

Total RNA was collected from targeted tissues or cells with the help of Trizol reagent (Invitrogen) followed by subsequent DNase I (Invitrogen) treatment following the protocols. cDNA was synthesized with the help of oligo (dT) 20 and Superscript II reverse transcriptase (Invitrogen). The expression of mRNA was examined using SYBR green PCR Master Mix (Qiagen, Venlo, Netherlands). The expression of miRNA was examined by a Hairpin-it TM miRNAs qPCR kit (Genepharma). The data were normalized to GAPDH (for mRNA expression) and RNU6B (for miRNA expression), respectively, and analyzed using a 2^-ΔΔCT^ method.

### Cell viability determination by MTT

Cell viability was detected using MTT (3-(4,5-dimethylthiazol-2-yl)-2,5-diphenyltetrazolium bromide) assays. The target cells were seeded in 96-well plates in a density of 5 × 10^3^ cells/well; 24 h later, target cells were subjected to transfection of shGal-3 or Gal-3 or si-NEAT1. Forty-eight hours later, the cells were co-incubated with 20 μl MTT (5 mg/ml; Sigma-Aldrich) in a humidified incubator for 4 h. Thereafter, 200 μl DMSO was used to dissolve the formazan after discarding the supernatant. The cell viability was then detected by reading the OD490 nm value normalizing to non-treated cells.

### DNA synthesis capacity by BrdU

The DNA synthesis capacity was detected by measurement of BrdU incorporation. A549 and H1299 cells (2 × 10^3^ cells/well) were seeded in 96-well plates and transfected and/or treated as described; twenty-four or forty-eight hours later, BrdU assays were performed. At the end of culturing, 10 μM BrdU (BD Pharmingen, San Diego, CA, USA) was added and the target cells were incubated for another two to twenty-four hours, when the medium was discarded and the cells were fixed by RT for 30 min. Then, peroxidase-coupled BrdU antibody (Sigma-Aldrich) was added and the target cells were incubated for 60 min. After washing by PBS and incubation with peroxidase substrate, the OD 450 nm value was examined. The value of non-exposed cells (incubated with BrdU antibody) was taken as background value.

### Migration capacity determination by Transwell assays

A total of 5 × 10^5^ target cells were transfected and/or treated as described, and seeded onto the upper chamber of polycarbonate Transwell filters (Cell Biolabs, Inc. Santiago, CA, USA) with no Matrigel nor serum at 37 °C for 48 h; medium with FBS was used in the bottom chamber. The non-migratory cells on the upper side were cleared away and the migratory cells on the lower side were counted under a microscope after fixing and staining with DAPI (Beyotime Institute of Biotechnology, Haimen, China).

### Immunoblotting (western blot, WB)

The protein levels of Galectin-3, TLR-4, MyD88, p-p65 and p65were examined by immunoblotting. The proteins were extracted from target cell lysate and analyzed for protein concentration using the bicinchoninic acid (BCA) protein assay kit (Beyotime Institute of Biotechnology). Extracted proteins were then loaded onto an SDS-PAGE minigel for separating. Separated proteins were then transferred onto PVDF membrane. Thereafter, the membrane was probed with the antibodies listed below, which were all obtained from Abcam (Cambridge, MA, USA) unless otherwise stated: Galectin-3 (mouse monoclonal, ab2785), TLR-4 (rabbit polyclonal, ab47093), MyD88 (rabbit polyclonal, ab2064), p-p65 (rabbit polyclonal, ab86299) and GAPDH (mouse monoclonal, ab8245) at 4 °C overnight. Thereafter, the blots were incubated with HRP-conjugated secondary antibody (1:5000). Signals visualization was conducted by ECL Substrates (Millipore, MA, USA) normalizing to GAPDH. The gray intensity analysis was performed using ImageJ software (NIH).

### Chromatin immunoprecipitation (ChIP)

After treating with LPS (20 ng/ml) for four hours, ChIP assay was performed according to previously described method [24] using anti-p65 (anti-p65, ab16502). The fold-enrichment (FE) of antibody binding DNA was calculated as described [24].

### P65-NEAT1 binding validation using luciferase activity

Two kinds of NEAT1 promoter luciferase reporter vectors were constructed: a wild-type containing wild-type NEAT1 promoter with predicted p65 responsive element (p65 RE), and a mutant-type containing a mutation in both or any of the putative binding sites in p65RE. HEK293 cell was used as a cellular tool to examined the alterations of luciferase activity 48 h after transfection of reporter vectors in the presence or absence of LPS, respectively using Dual Luciferase Reporter Assay System (Promega, Fitchburg, WI, USA).

### HE staining and immunohistochemistry

Lung adenocarcinoma tissues and the matched adjacent normal tissues were fixed in 10% formalin overnight and then processed by paraffin embedding and sectioning. Sections with a thickness of 5 μm were deparaffinized and rehydrated. Sections were then stained using HE staining kit (Beyotime, China) following the protocols. Immunohistochemical staining for Galectin-3 and TLR4 in lung adenocarcinoma tissues was performed as described previously [25] using Galectin-3 (ab2785) or TLR4 (ab47093) antibodies. The HE and immunohistochemical sections were then observed under an optical microscope (Olympus, Tokyo, Japan).

### Statistical analysis

Each experiment was repeated at least three times. Data were processed by SPSS 17.0 (SPSS, Chicago, IL, USA) and exhibited as mean ± standard deviation (SD). Differences between paired samples were compared using the Student’s paired test. The differences of more than two groups were evaluated using the one-way ANOVA. Kaplan-Meier analysis and the log-rank test were used to analyze the overall survival curves in patients with lung cancer. COX risk proportional regression model (univariate analysis and multivariate analysis) was employed to identify the risk factors for survival in patients with lung cancer. *P* values of < 0.05 were considered statistically significant.

## Results

### NEAT1 expression in lung adenocarcinoma tissue samples and its correlation with overall survival

**Fig. 1:**
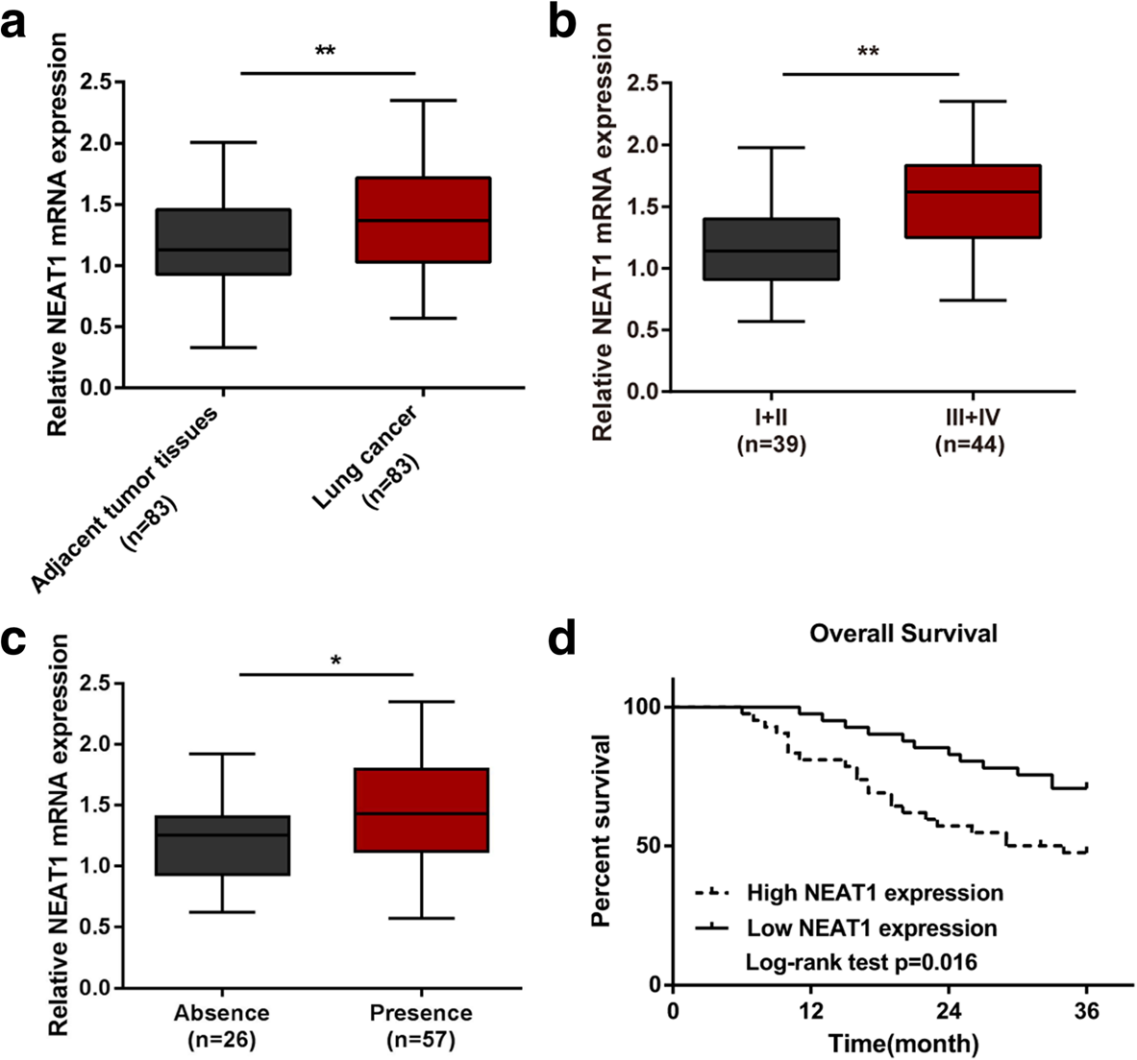
The role of NEAT1 in the regulation of lung cancer cell proliferation (**a**) The expression level of NEAT1 in 83 paired lung adenocarcinoma tissues and adjacent normal tissues was determined using real-time PCR assays. **b-c** NEAT1 expression in 83 cases of lung adenocarcinoma tissues was analyzed according to TNM staging and lymph node metastasis. The data are presented as mean ± SD of three independent experiments. *P < 0.05, **P < 0.01. (**d**) Kaplan-Meier overall survival curves for 83 patients with lung adenocarcinoma classified according to relative NEAT1 expression level

NEAT1 is abnormally expressed in lung cancer and acts as an oncogene [9, 10, 13]; herein, we first validated NEAT1 expression in 83 paired lung adenocarcinoma and adjacent normal specimens, as well as its correlation with the overall survival in patients with lung adenocarcinoma. As shown by real-time PCR, NEAT1 expression was dramatically increased in cancer tissue specimens compared to normal control (Fig. 1a); further analysis indicated that higher NEAT1 expression was more commonly observed in specimens in advanced TNM stages (Fig. 1b) and with lymph node metastasis (Fig. 1c). Patients were grouped as follows: a high NEAT1 group possessing NEAT1 expression higher than the median level and a low NEAT1 group possessing NEAT1 expression lower than the median level; the overall survival of patients in high NEAT1 group was remarkably shorter than that in the low NEAT1 group (Fig. 1d, *P* = 0.016). Moreover, higher NEAT1 expression was correlated with advanced TNM stages (*P* = 0.031, Table 1). The association between overall survival and pathological characteristics was analyzed then using a COX risk proportional regression model. Univariate analysis revealed that the difference in overall survival caused by tumor size, TNM stage or NEAT1 expression was statistically significant; multivariate analysis revealed that NEAT1 expression (Table 2) and TNM stages both represented independent factors related to the overall survival of patients involved (Table 2). The data reveal that NEAT1 expression is dysregulated in lung adenocarcinoma; its high expression is associated with shorter overall survival of patients with lung adenocarcinoma.

**Table 1:** Correlation between lncRNA NEAT1 expression and clinicopathological features in lung cancer patients

Characteristics		NEAT1 expression		*p*-value
		HIGH	LOW	
Age (years)	< 50	27	21	0.228
	≥ 50	15	20	
Gender	female	13	15	0.587
	male	29	26	
Tumor size(cm)	< 5	22	29	0.086
	≥ 5	20	12	
Histology	Adenoma	23	18	0.322
	Squamous	19	23	
Differentiation	Moderate-Poor	28	20	0.099
	Well	14	21	
TNM stage	I	5	9	0.031
	II	8	17	
	III	18	10	
	IV	11	5	
Lymph node metastasis	Absence	10	16	0.135
	Presence	32	25

**Table 2:** Univariate and multivariate analysis for factors related to overall survival using the COX proportional hazard model

Characteristics		Univariate analysis			Multivariate analysis		
		p	HR	95%CI	p	HR	95%CI
Age	< 50 vs ≥50	0.408	1.346	0.666–2.721	N.A		
Gender	female vs male	0.685	0.862	0.420–1.769	N.A		
Tumor size	< 5 vs ≥5	0.012	0.420	0.214–0.825	0.198	0.597	0.272–1.310
Histology	Adenoma vs Squamous	0.603	0.836	0.426–1.640	N.A		
Differentiation	Moderate-Poor vs Well	0.512	1.265	0.626–2.558	N.A		
TNM stage		< 0.001			0.002		
	I	0.008	0.242	0.085–0.692	0.161	0.416	0.122–1.417
	II	< 0.001	0.094	0.030–0.293	< 0.001	0.123	0.038–0.399
	III	0.002	0.294	0.133–0.649	0.003	0.290	0.127–0.664
	IV						
Lymph node metastasis	Absence vs Presence	0.426	1.363	0.636–2.920	N.A		
NEAT1	high vs low	0.018	2.339	1.156–4.733	0.035	2.199	1.055–4.581

### NEAT1 knockdown inhibits lung cancer cell proliferation and migration

**Fig. 2:**
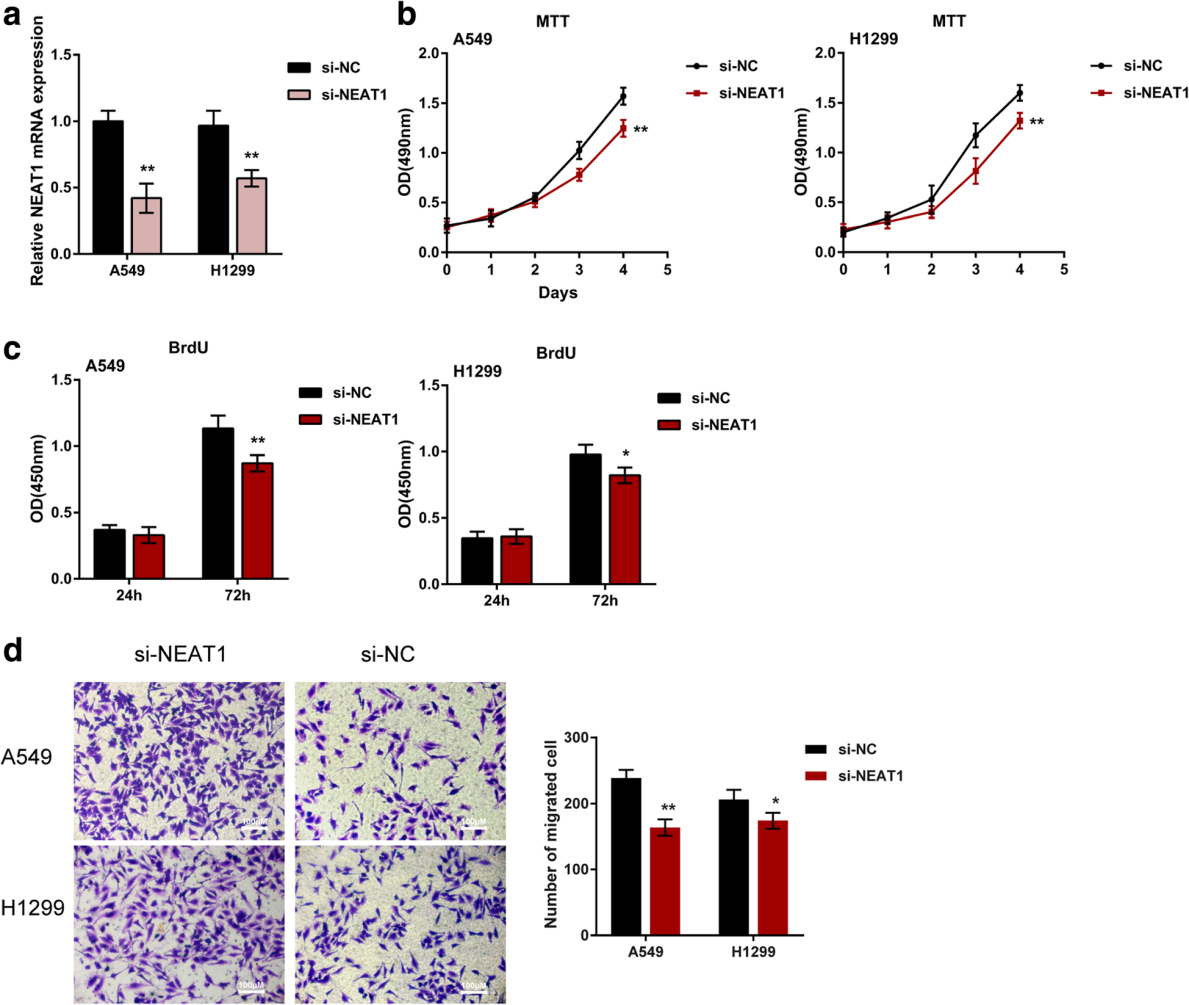
The effect of NEAT1 on lung cancer cell proliferation and migration (**a**) A549 and H1299 cells were transfected with si-NEAT1 to achieve NEAT1 knockdown, as confirmed using real-time PCR assays. **b-c** The proliferation of lung cancer cells was determined using MTT and BrdU assays. **d** The migration of lung cancer cells was determined using Transwell assays. The data are presented as mean ± SD of three independent experiments. *P < 0.05, **P < 0.01

In order to evaluate the detailed function of NEAT1 in cancer development, NEAT1 knockdown was conducted by transfecting si-NEAT1 into A549 and H1299 cells, as confirmed by real-time PCR (Fig. 2a). Next, the cell viability and DNA synthesis capacity of non-transfected or transfected tumor cells were measured by MTT and BrdU. After NEAT1 knockdown, the cell viability and DNA synthesis capacity of A549 and H1299 was significantly suppressed (Fig. 2b-c), indicating that NEAT1 knockdown could inhibit lung cancer cell proliferation. Moreover, NEAT1 knockdown also suppressed the migration capability of lung cancer cell (Fig. 2d). Consistent with previous studies, NEAT1 may contribute to lung cancer cell proliferation and migration; thus, inhibiting aberrant NEAT1 overexpression in lung cancer represents a promising strategy for regulating cancer cell growth.

### NF-κB binds to NEAT1 promoter region after LPS-stimulated p65 nucleus translocation

**Fig. 3:**
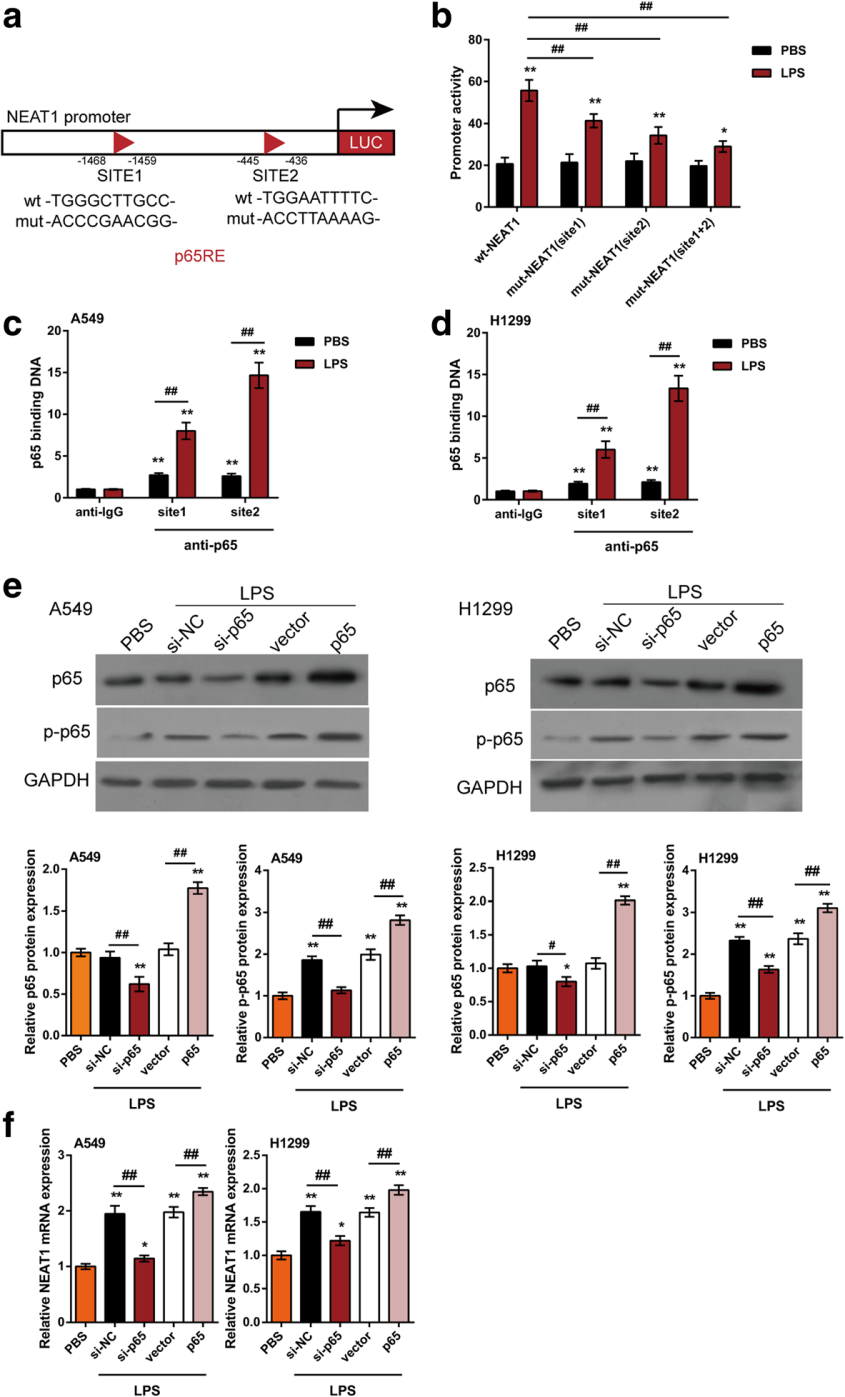
NF-κB binds to the promoter region of NEAT1 after LPS-stimulated p65 nucleus translocation (**a**) A schematic diagram of potential p65 binding element (two possible binding sites) in the promoter region of NEAT1 predicted by Jaspar database. A wt-NEAT1 promoter luciferase reporter vector and a mut-NEAT1 promoter luciferase reporter vector were constructed by mutating any or both of the predicted binding sites. **b** The indicated vectors were transfected into HEK293 cells and then treated with PBS or LPS (20 ng/ml for 4 h); the luciferase activity was determined. **c-d** The real-time ChIP assay showed that the level of p65 antibody binding to NEAT1 promoter was much greater than that of IgG. **e** A549 and H1299 cells were transfected with si-p65 or p65 vector; the protein levels of p65 and p-p65 were determined using Immunoblotting in the presence or absence of LPS (20 ng/ml for 4 h). **f** The mRNA expression of NEAT1 was determined using real-time PCR assays. The data are presented as mean ± SD of three independent experiments. *P < 0.05, **P < 0.01, compared to PBS group; #*P* < 0.05, ##*P* < 0.01, compared to si-NC or vector group

The activation of NF-κB, a key transcription factor which is constitutively activated in many cancers, is a crucial contributor in cancer progression [19, 20]. NF-κB can activate the expression of proliferation-, apoptosis- and/or migration-related genes [14, 16, 26] after multi factor-stimulated p65 nucleus translocation, including TNF-α, IL-1 and LPS [21]. Herein, we investigated whether LPS stimulates p65 nucleus translocation and subsequent NEAT1 expression activation. First, we validated the predicted binding between NF-κB and NEAT1 promoter region using luciferase reporter gene assays. Jaspar [27] database predicted that there might be two p65RE in NEAT1 promoter (p65RE, Fig. 3a). Two different types of luciferase reporter vectors, a wild-type NEAT1 (named wt-NEAT1, possessing no mutation) and a mutant-type NEAT1 (named mut-NEAT1, containing any or both of the mutated binding sites) were constructed (Fig. 3a). The vectors were transfected into HEK293 cells in the presence of PBS or LPS, which has been reported to stimulate p65 nucleus translocation. Afterwards, the luciferase activity was examined. LPS treatment significantly amplified the luciferase activity of wt-NKILA, comparing to PBS treatment. When any or both of the two putative binding sites were mutated, LPS-induced luciferase activity alterations were eliminated (Fig. 3b). Moreover, as shown by ChIP assay, the levels of p65 antibody binding to any of the predicted sites in NEAT1 promoter region were higher than those binding to IgG (Fig. 3c-d), suggesting that p65 may bind to the predicted binding site 1 or site 2 within NEAT1 promoter to activate its expression.

Further, A549 and H1299 cells were transfected with si-p65 or p65 vector in the presence of PBS or LPS stimulation; the protein levels of p65 and p65, as well as the mRNA expression of NEAT1 were determined. LPS stimulation caused no obvious changes in p65 protein levels in si-NC (negative control for si-p65) or empty vector (negative control for p65 vector) transfected cancer cells; however, the protein levels of p-p65 were strongly induced by LPS stimulation (Fig. 3e). P65 and p-p65 proteins were reduced by si-p65 while increased by p65 vector transfection; the inducible effect of LPS on p-p65 protein could be partially reversed by si-p65 while even amplified by p65 vector transfection (Fig. 3e). In addition, LPS stimulation remarkably up-regulated NEAT1 expression; si-p65 transfection partially attenuated, while p65 vector transfection even amplified the promotive effect of LPS on NEAT1 expression as shown by real-time PCR (Fig. 3f). The data suggest that LPS promotes p65 nucleus translocation, followed by activation of NEAT1 expression in lung cancer cells.

### TLR4 signaling is involved in NF-κB-mediated NEAT1 expression activation

**Fig. 4:**
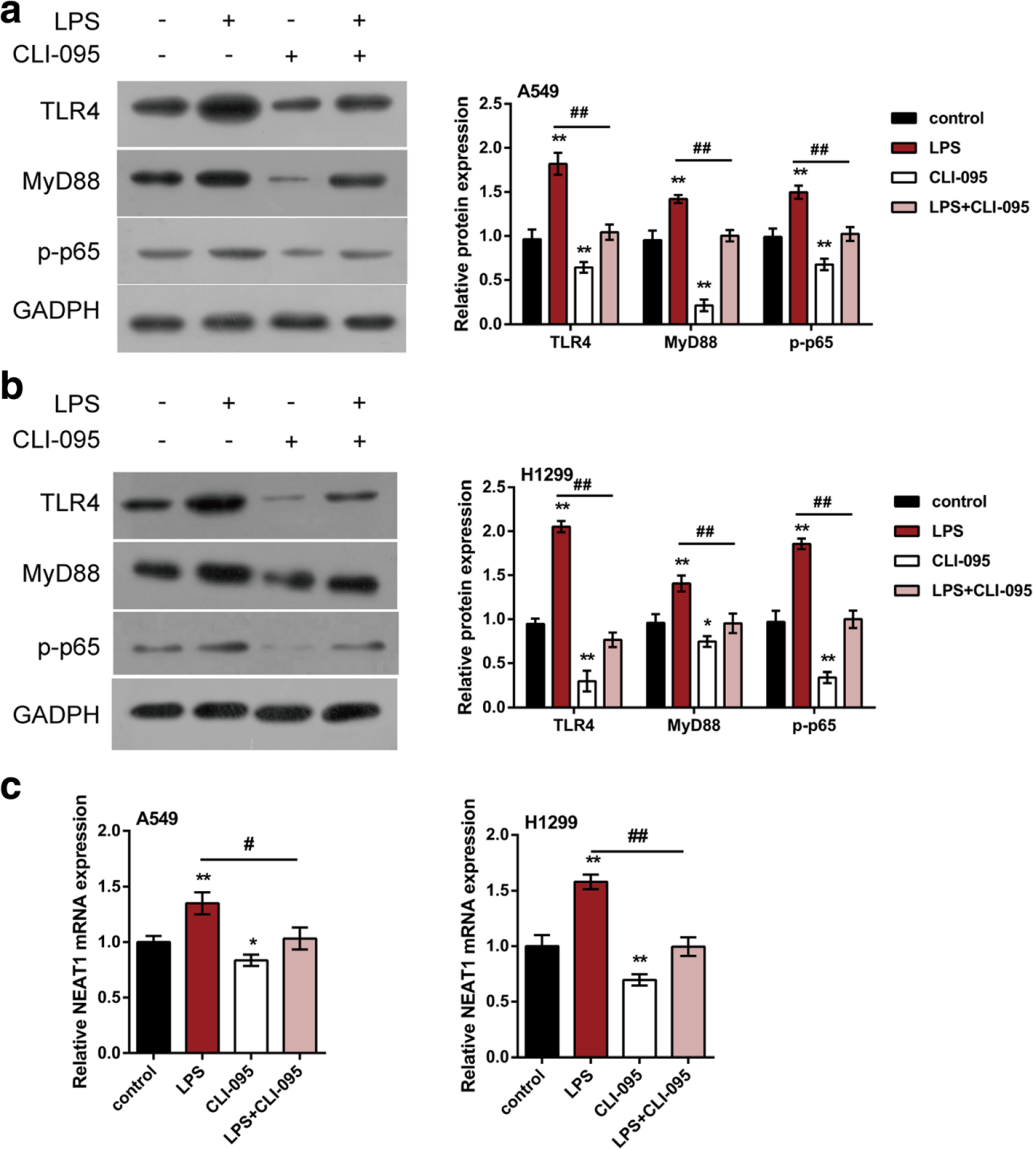
TLR4 signaling is involved in NF-κB-mediated NEAT1 expression activation (**a-b**) A549 and H1299 cells were co-treated with LPS (20 ng/ml) and CLI-095 (50 nmol/L) for 4 h; the protein levels of TLR4, MyD88 and p-p65 were determined using Immunoblotting assays. **c** The mRNA expression of NEAT1 was determined using real-time PCR assays. The data are presented as mean ± SD of three independent experiments. *P < 0.05, **P < 0.01, compared to control group; #*P* < 0.05, ##*P* < 0.01, LPS group compared to LPS + CLI-095 group

In addition to inducing p65 nucleus translocation, LPS has also been reported to activate TLR4 signaling, which is also an essential pathway in the pathopoiesis of tumors [28]; interestingly, TLR4/NF-κB signaling mediates diverse tumor growth [29, 30]. Herein, we investigated whether TLR4 is another component of the upstream regulatory signaling of NEAT1 expression in lung cancer cell lines. A549 and H1299 cells were treated with LPS, known to activate TLR4 signaling, and/or 50 nmol/L CLI-095, an inhibitor or TLR4 signaling; TLR4, MyD88 and p-p65 protein expression and NEAT1 mRNA expression. In LPS group, the protein levels of TLR4, MyD88 and p-p65 and NEAT1 mRNA expression were all up-regulated (Fig. 4a-c); on the contrary, CLI-095 caused an obvious suppression of TLR4, MyD88 and p-p65 protein expression and NEAT1 mRNA expression (Fig. 4a-c). The data indicated that LPS stimulation activates TLR4 signaling, followed by downstream NF-κB activation, and ultimately NEAT1 expression activation.

### Galectin-3 induced TLR4 signaling activation in lung cancer cells

**Fig. 5:**
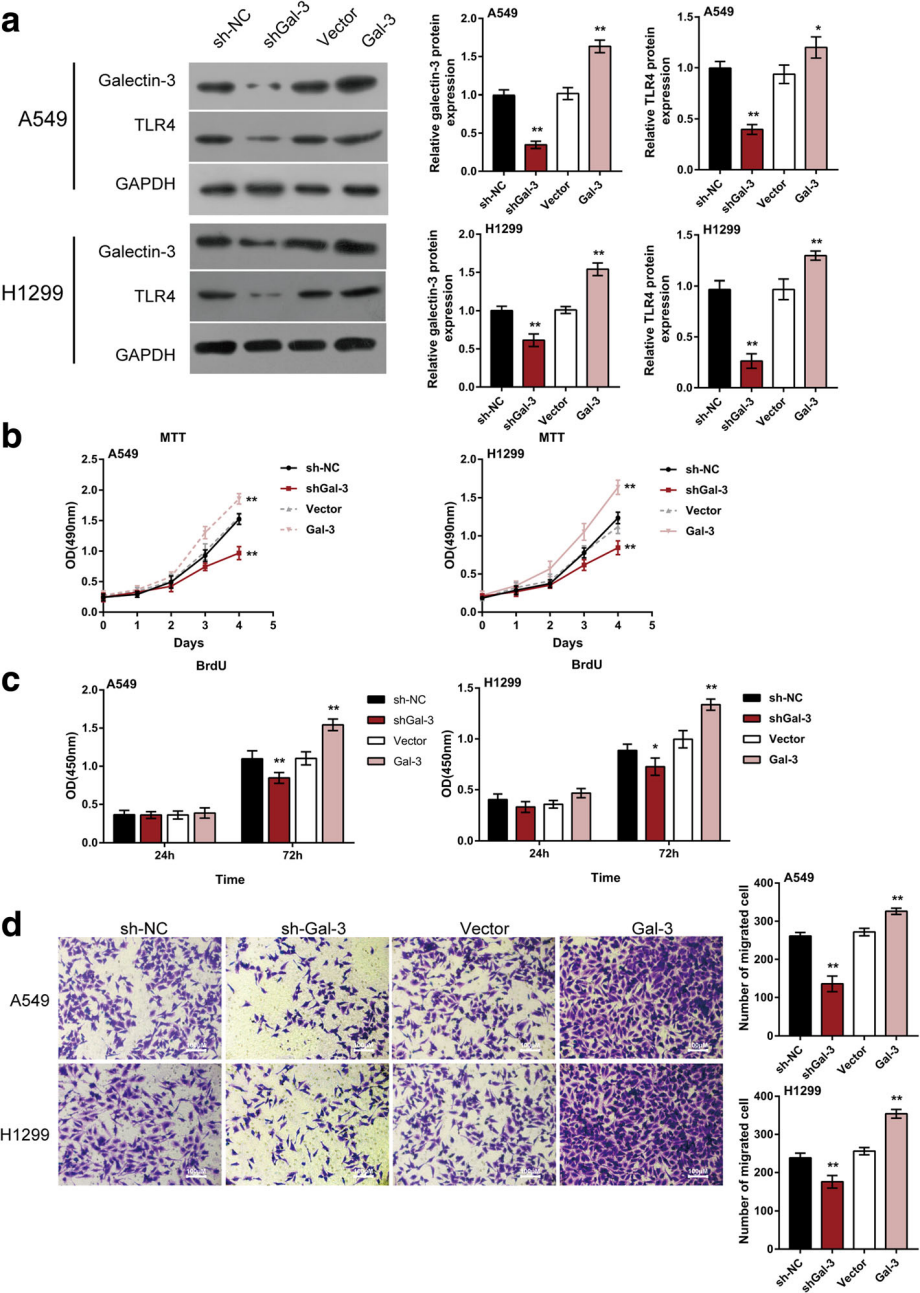
Galectin-3 induced TLR4 signaling activation in lung cancer cells (**a**) A549 and H1299 cells were transfected with shGal-3 or Gal-3 vector; the protein levels of Galectin-3 and TLR4 were determined using Immunoblotting assays. **b-c** The proliferation of lung cancer cell was determined using MTT and BrdU assays. **d** The migration of lung cancer cell was determined using Transwell assays. The data are presented as mean ± SD of three independent experiments. *P < 0.05, **P < 0.01, compared to sh-NC or Vector group

As we have demonstrated, TLR4/NF-κB/NEAT1 can be activated by LPS stimulation; are there any other dysregulated factors in lung cancer that can modulate this TLR4/NF-κB/NEAT1 axis? Galectin-3 has been regarded as a sensor-regulator in TLR pathways in synovial fibroblasts [31]; moreover, it has been regarded as a ligand of TLR4 and could promote TLR4, MyD88 and p-p65 expression [32, 33]. The overexpression of Galectin-3 has been observed in lung cancer [34, 35]. Herein, we hypothesized that Galectin-3 may activate TLR4/NF-κB/NEAT1, thereby affecting lung cancer cell proliferation and migration. To validate this hypothesis, A549 and H1299 cells were transfected with shGal-3 or Gal-3 vector to achieve Galectin-3 knockdown or overexpression, as confirmed by WB (Fig. 5a); TLR4 protein was positively regulated by Galectin-3 (Fig. 5a). Moreover, the cell proliferation and migration of lung cancer cell were significantly suppressed by Galectin-3 knockdown while promoted by Galectin-3 overexpression (Fig. 5b-d), indicating the potential role of Galectin-3 in activating TLR4 signaling and promoting lung cancer progression.

### Galectin-3 affects the proliferation and migration of lung cancer cell lines through TLR4/NF-κB/NEAT1

**Fig. 6:**
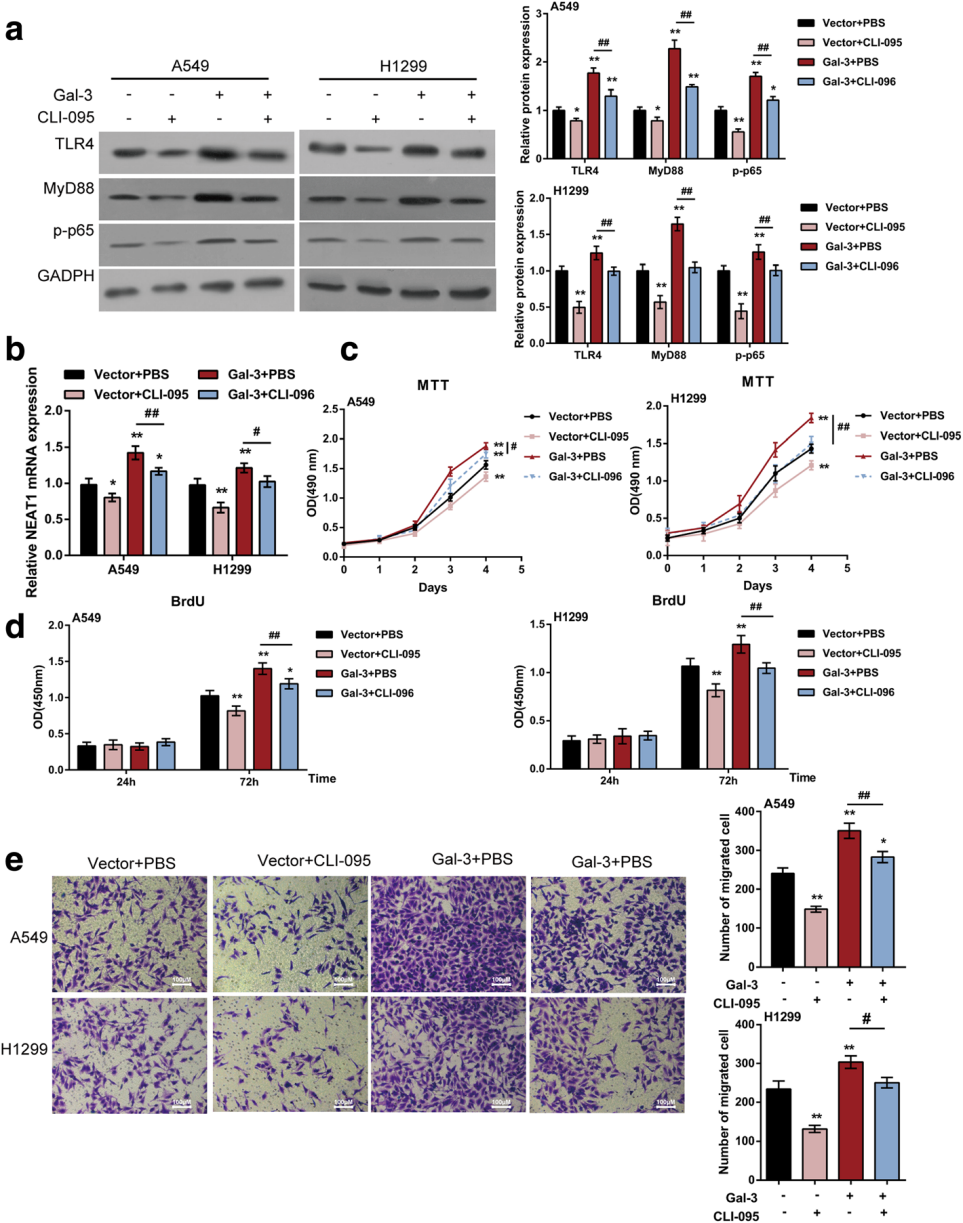
Galectin-3 affects lung cancer cell proliferation and migration through TLR4/NF-κB/NEAT1 (**a**) A549 and H1299 cells were transfected with Gal-3 in the presence or absence of CLI-095(50 nmol/L for 4 h); the protein levels of TLR4, MyD88 and p-p65 were determined using Immunoblotting assays. **b** The mRNA expression of NEAT1 was determined using real-time PCR assays. **c-d** The proliferation of lung cancer cell was determined using MTT and BrdU assays. **e** The migration of lung cancer cell was determined using Transwell assays. The data are presented as mean ± SD of three independent experiments. *P < 0.05, **P < 0.01, compared to control group; #*P* < 0.05, ##*P* < 0.01, Gal-3 group compared to Gal-3 + CLI-095 group

Given that Galactin-3 positively regulates TLR4 protein expression, as well as the proliferation and migration of lung cancer cell lines; next, we investigated whether Galectin-3 acts through TLR4/NF-κB/NEAT1. A549 and H1299 cells were transfected with Gal-3 in the absence or presence of 50 nmol/L CLI-095; the protein levels of TLR4, MyD88 and p-p65, and NEAT1 mRNA expression were determined. The results showed that CLI-095 dramatically decreased the protein levels of TLR4, MyD88 and p-p65, and NEAT1 mRNA expression; on the contrary, Gal-3 transfection increased TLR4, MyD88 and p-p65 proteins and NEAT1 mRNA expression; moreover, the inhibitory effect of CLI-095 on the above factors could be partially abolished by Gal-3 transfection (Fig. 6a-b).

Furthermore, we also monitored the changes of lung cancer cell proliferation and migration under the same conditions. Consistent with above results, Galectin-3 overexpression significantly promoted, whereas CLI-095 remarkably suppressed the cell proliferation and migration of lung cancer cell lines; the above effects of Galectin-3 were partially eliminated by CLI-095 (Fig. 6c-e), indicating that Galectin-3 activates TLR4 signaling, followed by downstream NF-κB activation and NEAT1 expression upregulation, finally resulting in excessive proliferation and migration of tumor cells.

### The expression levels of Galectin-3 and TLR4 in lung adenocarcinoma tissue samples and their correlation with NEAT1

**Fig. 7:**
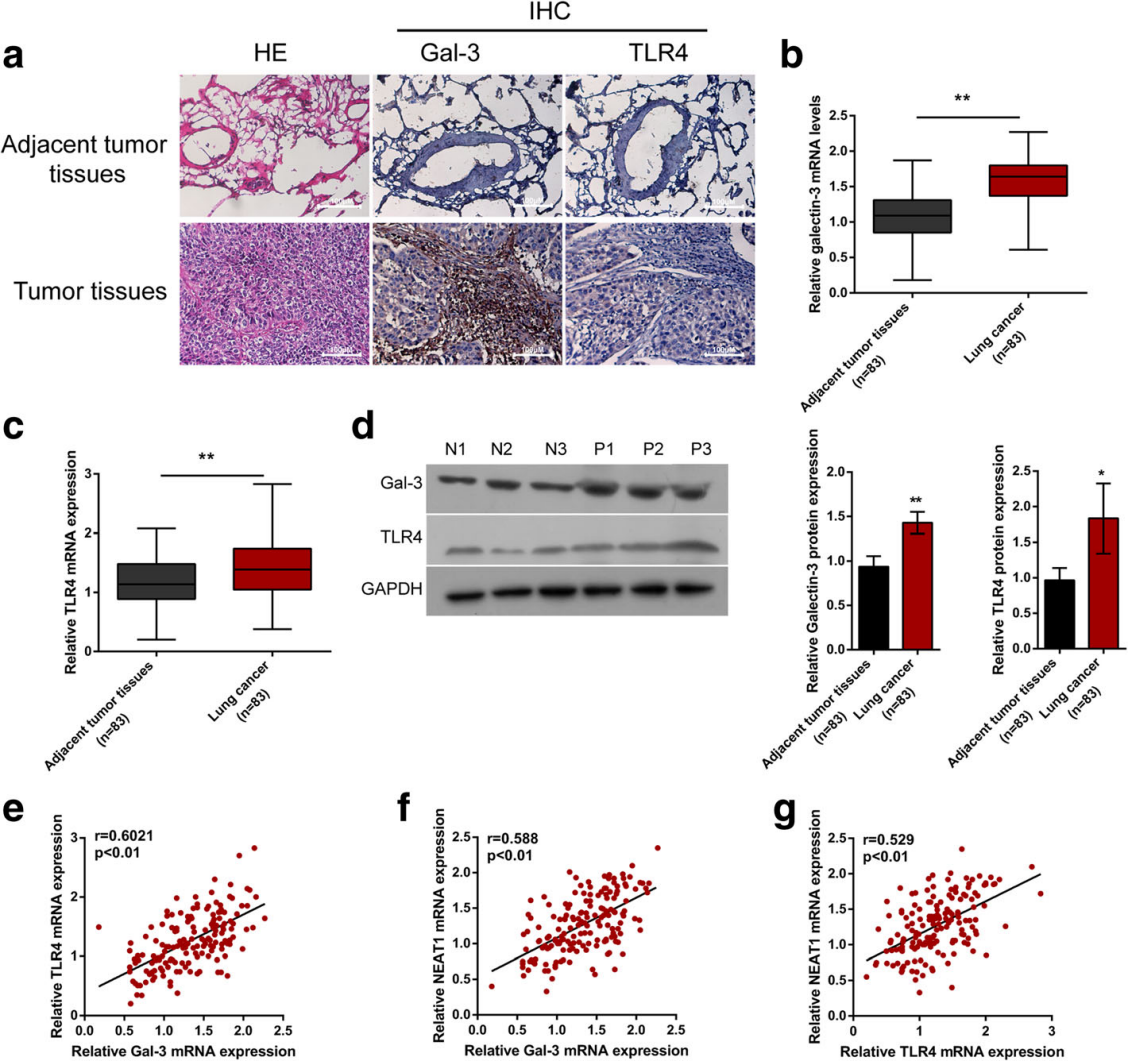
The expression levels of Galectin-3 and TLR4 in lung adenocarcinoma tissues and their correlation with NEAT1 (**a**) Pathological morphologic changes of lung adenocarcinoma tissues and adjacent normal tissues were shown by H&E staining; Gal-3 and TLR4 expression in lung adenocarcinoma tissues and adjacent normal tissues were shown by IHC staining. **b-d** The mRNA and protein expression of Galectin-3 and TLR4 in lung adenocarcinoma tissues and adjacent normal tissues was determined using real-time PCR and Immunoblotting assays. The data are presented as mean ± SD of three independent experiments. *P < 0.05, **P < 0.01. **e-g** The correlation of Galectin-3, TLR4 and NEAT1 was analyzed using Spearman’s rank correlation analysis

As a further confirmation of these data, the mRNA expression of Galectin-3 and TLR4 in lung adenocarcinoma and non-cancerous tissue samples were examined. As shown by H&E staining, in lung adenocarcinoma tissues, the cancer cells were irregularly arranged and the nucleus shapes of the nucleus were shown (Fig. 7a). Galectin-3 and TLR4 were both overexpressed in lung adenocarcinoma tissues, compared to those in non-tumor control (Fig. 7a). Moreover, the mRNA expression and protein levels of Galectin-3 and TLR4 in lung adenocarcinoma samples were much higher than those in normal lung tissues (Fig. 7b-d). Moreover, a positive correlation between Galectin-3 and TLR4 mRNA, between Galectin-3 and NEAT1, between TLR4 and NEAT1 was observed (Fig. 7e-g). The data indicate that Galectin-3 and TLR4 expression are abnormally up-regulated in lung adenocarcinoma tissues; Galectin-3 affects lung adenocarcinoma cell proliferation and migration through activating TLR4/NF-κB pathway and NEAT1 expression.

## Discussion

**Fig. 8:**
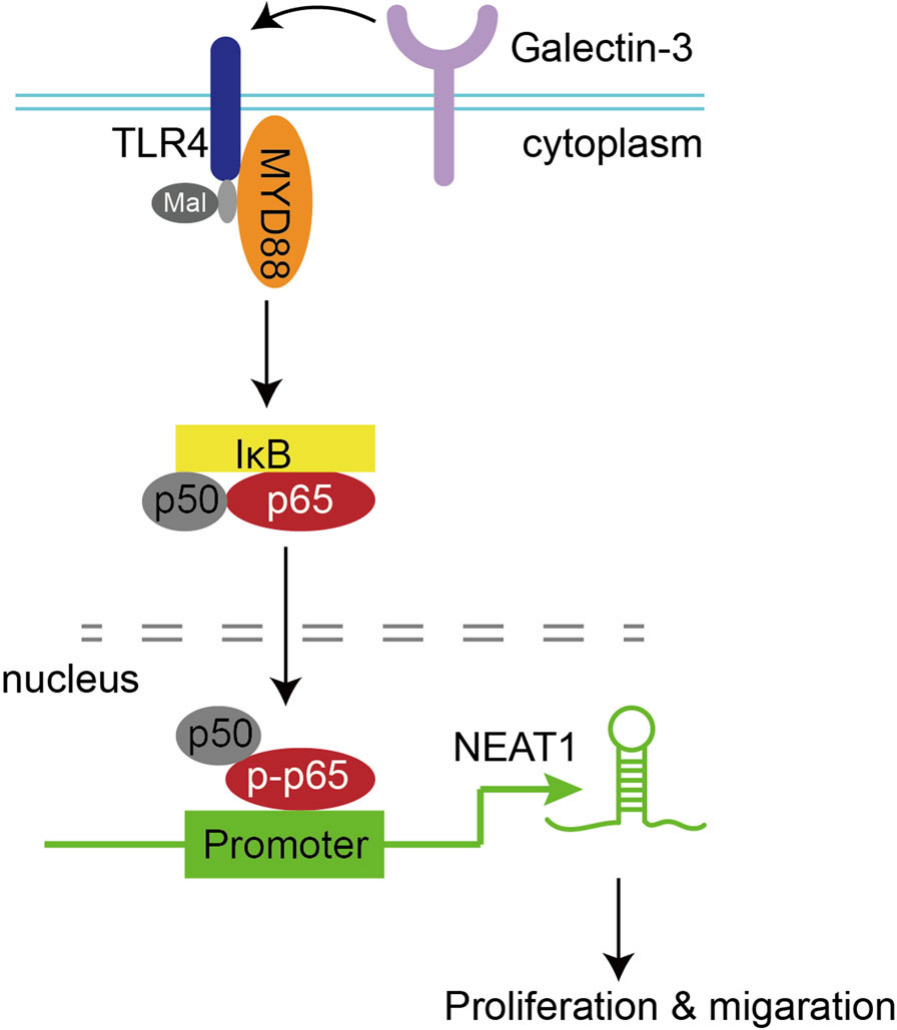
Galectin-3-induced TLR4/NF-κB signaling activation contributes to lung adenocarcinoma cell proliferation and migration through activation of p65 nucleus translocation and NEAT1 expression upregulation

In the present study, we reported abnormal lncRNA-NEAT1 overexpression in lung adenocarcinoma specimens; NEAT1 knockdown could inhibit lung cancer cell proliferation and migration. Furthermore, we demonstrated the upstream regulatory mechanism of NEAT1 disorder in lung adenocarcinoma, that is, Galectin-3 activates TLR4/NF-κB signaling, followed by p65 nucleus translocation, ultimately activating NEAT1 expression and promoting lung adenocarcinoma cell proliferation and migration (Fig. 8).

The essential role of lncRNA in cancers has been frequently reported. Dysfunction of lncRNA can make contribution to cancer cell hyperproliferation, invasion and metastasis. The abnormal upregulation of NEAT1, a newly described lncRNA, has been observed in many cancers and play critical roles in tumorigenesis [36–38]. Higher NEAT1 expression is positively correlated with patient age, vascular invasion, lymphatic metastasis and TNM staging [13]. More importantly, NEAT1 expression is also up-regulated in lung cancer tissues or plasma [12, 13]; increased NEAT1 expression is related to unfavorable prognosis in patients with lung cancer, thus is regarded as a diagnosis marker [12]. Herein, we validated the increased NEAT1 expression in lung adenocarcinoma specimens, as well as the correlation between higher NEAT1 expression and shorter overall survival in patients. NEAT1 overexpression also promotes lung adenocarcinoma cell proliferation and migration; thereby, investigating the mechanism of abnormal NEAT1 overexpression and rectifying the dysregulation of NEAT1 may represent a promising strategy for lung adenocarcinoma treatment.

The activation of NF-κB, a key transcription factor which is constitutively activated in many cancers, is a crucial contributor in cancer progression [19, 20]. NF-κB can be activated by miR-675, which is overexpressed in NSCLC and promotes NSCLC progression [39]. In lung cancer cells, p65 nucleus translocation can be activated by TGF-β1, followed by E-cadherin upregulation and increased epithelial-mesenchymal transition [40]. NF-κB activates the expression of proliferation-, apoptosis- and/or migration-related genes [14, 16, 26] after p65 nucleus translocation induced by multi factors, including TNF-α, IL-1 and LPS [21]. Herein, we investigated whether abnormal NEAT1 overexpression is associated with NF-κB activation in lung adenocarcinoma. Combined with the results from luciferase reporter gene and ChIP assays, NF-κB could bind to NEAT1 promoter to activate its expression. Upon the stimulation of LPS, p-p65 protein and NEAT1 mRNA expression were both significantly increased; while these changes can be attenuated by p65 knockdown and enhance by p65 overexpression, indicating that LPS-induced NF-κB activation is associated with NEAT1 upregulation.

In addition to inducing p65 nucleus translocation, LPS also activates the expression of TLR4, a key factor in TLR4/NF-κB signaling pathway, another crucial pathway in the pathopoiesis of tumors [28]. LPS enhances the protein expression of TLR4 and p65 in rat pulmonary arterial smooth muscle cells [41]. Interestingly, TLR4/NF-κB signaling mediates diverse tumor growth [29, 30]. In lung adenocarcinoma, LPS stimulation also caused a remarkable increase in TLR4, MyD88 and p-p65 protein expression, as well as NEAT1 mRNA expression, which could be partially reversed by CLI-095, an inhibitor of TLR4 signaling. The data indicate that TLR4/NF-κB signaling activation promotes NEAT1 expression through NF-κB binding to NEAT1 promoter. Consistent with our data, in colon cancer, NF-κB can be induced by LPS binding to TLR4 [42], further indicating the essential role of TLR4/NF-κB pathway in cancer development. However, LPS-induced TLR4/NF-κB activation has been well studied in cancers; herein, we investigated other possible upstream factors which can activate this key signaling pathway in lung adenocarcinoma.

Galectins, a series of proteins also known as animal lectins, possess different biological activities. Galectins can participate in diverse cellular physiological activities through interaction with either cell-surface and extracellular matrix glycoproteins and glycolipids, or intracellular cytoplasmic and nuclear proteins. Galectin-3 can induce ovarian cancer cell survival and chemoresistance through activating TLR4 pathway [33]. We revealed that Galectin-3-induced TLR4 signaling activation is involved in miR-548c regulation of lung cancer cell proliferation (data not shown). Herein, we also observed that Galectin-3 overexpression remarkably induced TLR4 protein expression, as well as lung adenocarcinoma cell proliferation and migration; next, we further investigated whether Galectin-3 exerts its function through downstream NF-κB and NEAT1. Similar as LPS, Galectin-3 overexpression also caused a dramatical increase in TLR4, MyD88 and p-p65 proteins and NEAT1 mRNA expression, which could also be partially reversed by CLI-095; in addition, the promotive effects of Galectin-3 on lung adenocarcinoma cell proliferation and migration was also partially attenuated by CLI-095. The data indicate that Galectin-3 induces TLR4/NF-κB signaling activation, followed by NEAT1 expression upregulation, and ultimately promotes lung adenocarcinoma cell proliferation and migration. Finally, we revealed a higher expression of Galectin-3 and TLR4 in lung cancer tissues; Galectin-3, TLR4 and NEAT1 was positively correlated with each other, indicating that Galectin-3/TLR4/NF-κB/NEAT1 path might be another contributor to the excessive cancer cell proliferation and migration of lung adenocarcinoma. Interestingly, data from online database TCGA indicated that Galectin-3 or TLR4 expression was not significantly correlated with the overall survival of patients with lung adenocarcinoma (sample size = 343, Additional file 1: Figure S1); considering that lower NEAT1 expression was significantly correlated with longer overall survival, the significance of NEAT1 being a potential prognosis marker might be greater.

Regarding the shortcomings of the present study, the expression and function of Galectin-3 in healthy cell lines should be examined before the application of Galectin-3 to clinical use, in order to ensure the safety and efficiency. Moreover, due to the wide activation of NF-κB in cancers, the efficiency of Galectin-3/TLR4/NF-κB/NEAT1 path should be compared with NF-κB activation induced by other factors in vitro and in vivo.

## Conclusions

We demonstrated Galectin-3-induced TLR4/NF-κB signaling activation could contribute to lung adenocarcinoma cell proliferation and migration through p65 nucleus translocation and NEAT1 expression upregulation; Galectin-3/TLR4/NF-κB/NEAT1 path might be another contributor to the hyperproliferation and migration of lung adenocarcinoma cells.

## Additional file

Additional file 1: **Figure S1.** Data from online database TCGA analyzing the correlation of Galectin-3 (A and C) or TLR4 expression (B and D) with the overall survival of patients with lung adenocarcinoma. (TIF 1101 kb)

## Abbreviations

BCA: Bicinchoninic acid
ChIP: Chromatin immunoprecipitation
FE: Fold-enrichment
lncRNAs: Long non-coding RNAs
LPS: Lipopolysac-charide
MTT: 3-(4,5-dimethylthiazol-2-yl)-2,5-diphenyltetrazolium bromide
NC: Negative control
ncRNAs: Non-coding RNAs
NEAT1: Nuclear enriched abundant transcript 1
NSCLC: Non-small cell lung cancer
p65RE: P65 responsive element
SD: Standard deviation
WB: Western blot
