# Supplementary figures and images for: Galectin-3 activates TLR4/NF-κB signaling to promote lung adenocarcinoma cell proliferation through activating lncRNA-NEAT1 expression
Source: BMC Cancer. 2018 May 22;18:580. doi: 10.1186/s12885-018-4461-z (PMC5964910; doi:10.1186/s12885-018-4461-z)

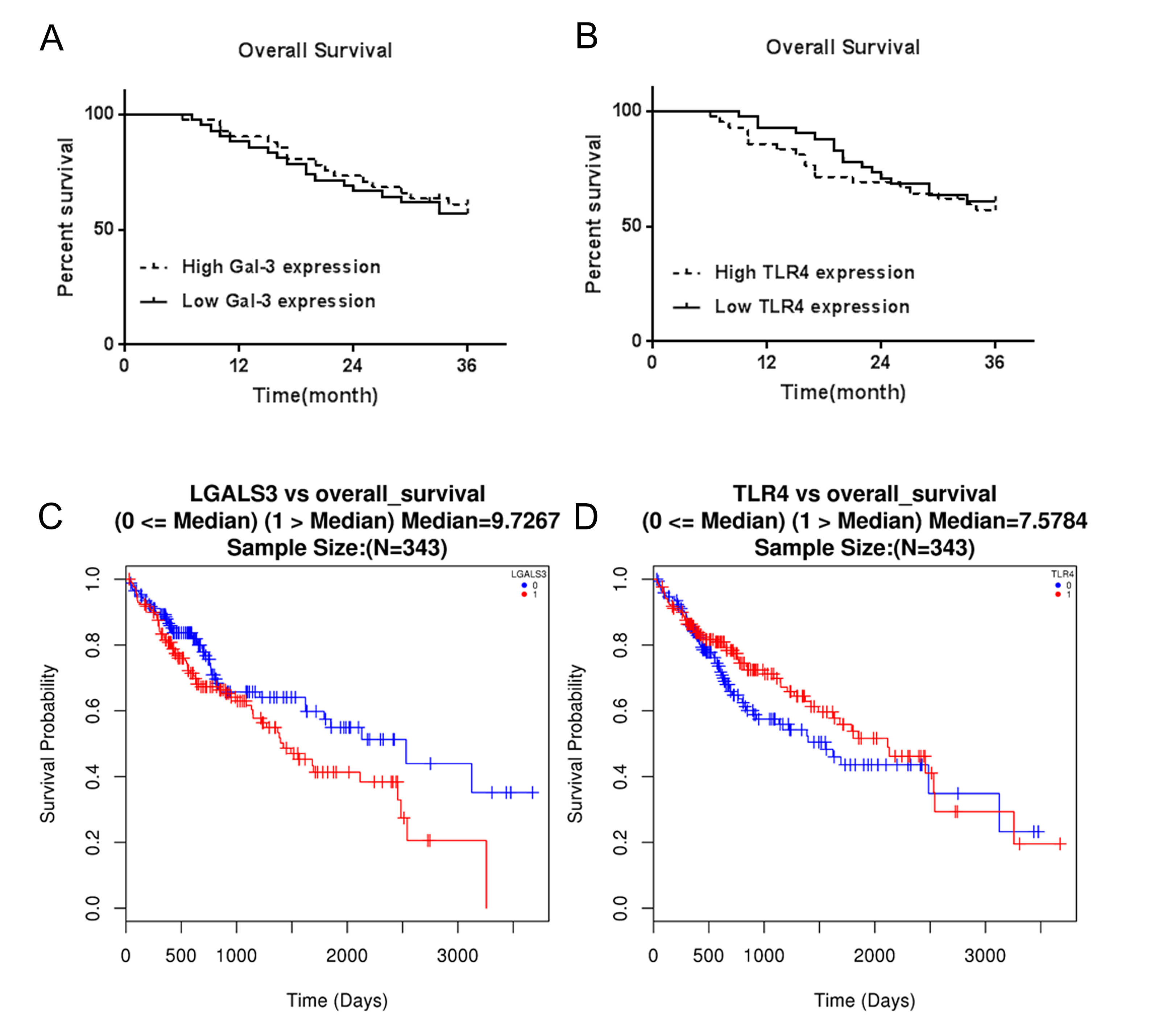

Supplement: Supplementary file 1 — Figure S1. Data from online database TCGA analyzing the correlation of Galectin-3 (A and C) or TLR4 expression (B and D) with the overall survival of patients with lung adenocarcinoma. (TIF 1101 kb) [file 12885_2018_4461_MOESM1_ESM.tif]
